# Off-season biogenic volatile organic compound emissions from heath mesocosms: responses to vegetation cutting

**DOI:** 10.3389/fmicb.2013.00224

**Published:** 2013-08-15

**Authors:** Riikka Rinnan, Diana Gierth, Merete Bilde, Thomas Rosenørn, Anders Michelsen

**Affiliations:** ^1^Terrestrial Ecology Section, Department of Biology, University of CopenhagenCopenhagen, Denmark; ^2^Center for Permafrost, University of CopenhagenCopenhagen, Denmark; ^3^Department of Physiology and Cell Biology, Molecular Plant Nutrition, Leibniz Institute of Plant Genetics and Crop Plant Research, Stadt SeelandGatersleben, Germany; ^4^Department of Chemistry, University of CopenhagenCopenhagen, Denmark

**Keywords:** induced volatiles, BVOC, sesquiterpenes, soil, plant wounding, grazing, *Deschampsia flexuosa*, arctic

## Abstract

Biogenic volatile organic compounds (BVOCs) affect both atmospheric processes and ecological interactions. Our primary aim was to differentiate between BVOC emissions from above- and belowground plant parts and heath soil outside the growing season. The second aim was to assess emissions from herbivory, mimicked by cutting the plants. Mesocosms from a temperate *Deschampsia flexuosa*-dominated heath ecosystem and a subarctic mixed heath ecosystem were either left intact, the aboveground vegetation was cut, or all plant parts (including roots) were removed. For 3–5 weeks, BVOC emissions were measured in growth chambers by an enclosure method using gas chromatography-mass spectrometry. CO_2_ exchange, soil microbial biomass, and soil carbon and nitrogen concentrations were also analyzed. Vegetation cutting increased BVOC emissions by more than 20-fold, and the induced compounds were mainly eight-carbon compounds and sesquiterpenes. In the *Deschampsia* heath, the overall low BVOC emissions originated mainly from soil. In the mixed heath, root, and soil emissions were negligible. Net BVOC emissions from roots and soil of these well-drained heaths do not significantly contribute to ecosystem emissions, at least outside the growing season. If insect outbreaks become more frequent with climate change, ecosystem BVOC emissions will periodically increase due to herbivory.

## Introduction

Ecosystem-level emissions of biogenic volatile organic compounds (BVOC) are considered to mainly originate from plant leaves (Laothawornkitkul et al., 2009). Some studies have identified that stems including bark (Sallas et al., 1999; Amin et al., 2012), plant roots and the rhizosphere (Bais et al., 2004; Lin et al., 2007), decomposing litter (Warneke et al., 1999; Leff and Fierer, 2008), and even microorganisms (Schulz and Dickschat, 2007; Korpi et al., 2009; Insam and Seewald, 2010) also release BVOCs contributing to the blend of compounds emitted from natural ecosystems. However, the emissions from soil and belowground plant parts (including roots and rhizomes), are still poorly characterized (Lin et al., 2007; Insam and Seewald, 2010).

The contribution of BVOCs to the carbon loss from soil is minimal relative to the respiratory CO_2_ effluxes (Aaltonen et al., 2011; Faubert et al., 2012). However, BVOCs are important as reactive atmospheric trace gases, and BVOC oxidation products contribute to secondary organic aerosol formation and may even be involved in new particle formation (see e.g., Fuentes et al., 2000; Jimenez et al., 2009; Riipinen et al., 2012). In addition to their role in atmospheric chemistry, BVOCs also play an important part in many biological interactions (Lehninh et al., 1999; Dicke and Bruin, 2001). In the soil atmosphere, BVOCs serve as a carbon source for some microorganisms, but they also have adverse effects on biogeochemical cycles (White, 1994; Smolander et al., 2006) and influence microbial activity, which can have important implications for ecosystem processes. For instance, monoterpenes have been observed to inhibit nitrogen mineralization, nitrification and methane oxidation, and stimulate carbon mineralization in soil (White, 1991; Amaral and Knowles, 1998; Smolander et al., 2006).

Separation of soil and vegetation emissions has been attempted in a few field studies. One of the first studies was conducted by Hayward et al. (2001), who used a dynamic chamber technique to measure monoterpene emissions from the forest floor and the foliage of a *Picea sitchensis* forest. Most of the forest floor emissions were reported to stem from needle litter and roots rather than from bulk soil (Hayward et al., 2001), although the potential soil emissions could not be separated from those of belowground plant material with the applied experimental strategy (removing soil layers). A field study conducted in a mountain birch forest in Abisko, northern Sweden compared emissions from vegetated forest floor plots to emissions from plots where aboveground vegetation had been removed by cutting (Faubert et al., 2012). The removal of the aboveground vegetation reduced the number of different BVOCs emitted whilst having no significant effects on the total quantity emitted, but again, it was not possible to separate emissions from soil and belowground plant parts.

Past research has temporally concentrated on the growing season period when biological activity is at its highest. However, recent studies have revealed that boreal forest floor BVOC emissions peak during early summer and autumn (Aaltonen et al., 2011) and not at midsummer even though the green plant biomass is peaking at midsummer. BVOC emissions can even be measured from the snowpack during winter (Helmig et al., 2009; Aaltonen et al., 2012). In this work we focus on BVOC emissions both from soil and the whole ecosystem in a period of the year which has hither-to been largely neglected, namely the shoulder periods between summer and winter.

Results from laboratory studies assessing BVOCs emissions from root-free soil and litter samples indicate that soil emissions are controlled by both microbial activity and substrate quality. Stahl and Parkin (1996) measured contrasting BVOC emission spectra from soils amended with different substrates and selective inhibitors. Leff and Fierer (2008) detected 100 different compounds, 70 of which were identified, in emissions from 40 different soil and litter samples. The emissions from the soil samples appeared to be related to the overall level of microbial activity in soil, while those from the litter samples were best predicted by the organic carbon quality (Leff and Fierer, 2008).

The main aim of this work was to differentiate between BVOC emissions from above- and belowground plant parts and soil outside of the growing season. We compared emissions from intact vegetation-soil mesocosms to emissions from mesocosms with belowground plant parts plus soil and further to emissions from root-free soil mesocosms. The mesocosms originated from two different heath ecosystems: (1) a subarctic heath with mixed vegetation dominated by evergreen dwarf shrubs and soil characterized by high soil organic matter content and (2) a semi-natural temperate heath with monospecific stands of the grass *Deschampsia flexuosa* and sandy soil. In both systems, the experiments were conducted with largely inactive vegetation to elucidate off-season BVOC emissions.

While many BVOCs are constitutively emitted by plants and other living organisms, their production can also be induced by abiotic (Loreto and Schnitzler, 2010) or biotic stresses (Holopainen and Gershenzon, 2010). In the experimental setup of the present study, we cut the aboveground vegetation to obtain mesocosms with only belowground plant material. This allowed us to estimate how mechanical damage affected the BVOC emissions from heath ecosystems. In nature, mechanical damage similar to that caused by cutting can occur via grazing, freezing or drying of plants. The *Deschampsia* heath of this work belongs to semi-natural ecosystem types that have been traditionally managed by grazing. Subarctic heaths are browsed by both large grazers, such as reindeer (*Rangifer tarandus*), and small rodents, like voles (*Clethrionomys rufocanus*) and lemmings (*Lemmus lemmus*). In addition, insect outbreaks shape the vegetation community.

With the help of the vegetation removal treatments we aimed to answer the following questions: What fraction of total BVOC emissions from heath ecosystems originates from belowground plant parts and what fraction originates from the soil alone outside of the growing season? Which compounds are emitted from vegetation and which from soil? Does vegetation cutting induce BVOC emissions from plants outside the main growing season?

## Methods

### Collection of mesocosm

Material for the experiment originated at a subarctic heath located in Abisko, Sweden (68°20′N, 18°50′E) and a temperate heath located in Brandbjerg, Denmark (55°53′N; 11°58′E). In both locations, nine mesocosms, quadrants of 20 × 20 cm with intact vegetation on top, were cut with a knife to the soil depth of 10 cm and mounted into an aluminium frame, which rested on a metal base in the growth chambers. The upper 0–10 cm soil contains ca. 76% of the total fine root biomass at the mixed heath (Rinnan, unpublished data) and ca. 71% of the total fine root biomass at the *Deschampsia* heath (Arndal, unpublished data).

The vegetation in the mesocosms from Abisko was dominated by Empetrum nigrum ssp. hermaphroditum and Rhododendron lapponicum and accompanied with Andromeda polifolia, Vaccinium uliginosum, Arctostaphylos alpina, Tofieldia pusilla, and Carex vaginata as minor components. The soil was highly organic (organic matter content 89 ± 1%), 10–15 cm deep, overlaying stones or bedrock, and had a pH of 6.8. These mesocosms were collected in late growing season, at the end of August 2010.

The mesocosms from Brandbjerg were collected from areas dominated by the perennial grass *Deschampsia flexuosa* in early November 2010. The soil consisted of 70% sand, 20% coarse sand, 6% silt, and 2% clay, and had a pH of about 4.2 in the organic layer and 3.5 in the upper mineral soil, and the soil organic matter content was 5.7 ± 0.4%.

All the mesocosms were transported to the University of Copenhagen campus by air freight (Abisko, 7 h) or surface transport (Brandbjerg, 1 h), where they were stored outdoors until the start of the experiment and watered if needed. The storage conditions in Copenhagen, with a mean temperature of 13.4°C in September 2010, exposed the mesocosms from Abisko to a longer than normal but fairly natural transition from growing season to autumn.

### Vegetation manipulations and analyses

**Figure 1:**
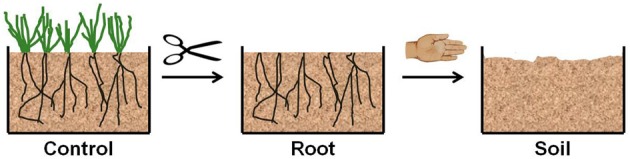
**An illustration of the experimental manipulations of heath mesocosms.** The control mesocosms had intact vegetation. In the root mesocosms all aboveground vegetation was cut with scissors. In the soil mesocosms aboveground vegetation was cut and roots were removed by hand-sorting.

The mesocosms from Abisko and Brandbjerg were treated in a similar manner (Figure 1): The vegetation in three mesocosms was left intact and these served as control mesocosms. In three mesocosms all aboveground vegetation was cut with scissors (from here on “root mesocosms”) and in another three the cutting of aboveground vegetation was followed by removal of roots and rhizomes from the soil (“soil mesocosms”). The belowground plant parts were removed from each mesocosm by hand-sorting through the soil for an equal amount of time.

At the end of the growth chamber experiment, above- and belowground vegetation from all mesocosms was collected and the soil was sorted as described above. The aboveground plant parts were separated into species and into leaves and stems when applicable. All vegetation samples were dried for 72 h at 70°C to obtain dry biomass.

### Growth chamber conditions

Two separate experiments were carried out, one with the mesocosms from Abisko on October 18–November 22, 2010 and one with the mesocosms from Brandbjerg on December 3–23, 2010. The mesocosms were divided into three growth chambers with air circulation, each with one mesocosm from each of the three treatments, yielding three replicate mesocosms per treatment. The conditions in the growth chambers were set to represent a shoulder season between summer and winter, taking into account that the temperature at the level of vegetation in low-stature systems is up to 10°C higher than air temperature at 2 m height (Scherrer and Körner, 2010). During the experiment, the mean 24-h air temperature at the level of vegetation was 13.6 and 18.0°C for the Abisko and Brandbjerg mesocosms, respectively. The daylight lasted 16 h and the darkness 8 h, and the daylight PAR at the level of vegetation was 200–300 μE s^−1^ m^−2^. The soil water content was maintained at 40%.

### VOC sampling and analysis

The mesocosms were sampled for VOC emissions before the vegetation manipulation treatments, right after performing the treatments and at intervals after that. During the sampling, the growth chamber door was closed, which ensured that light, temperature, and humidity stayed constant.

VOCs were sampled by a push-pull system described by Faubert et al. (2010, 2012) for 30 min. A transparent polycarbonate chamber (23 × 23 cm, height 25 cm), equipped with a fan to mix the headspace air, was placed on top of a water-filled groove in the aluminum frame holding each mesocosm. The water-filled groove sealed up the connection from the chamber to the aluminum frame. Pumps (12 V, Rietschle Thomas, Puchheim, Germany) pushed air through a charcoal filter and a MnO_2_ scrubber, to remove hydrocarbon impurities and ozone, respectively, into the chamber with a flow rate of 215 ml min^−1^. At the same time, air was pulled from the chamber with a flow rate of 200 ml min^−1^ through a stainless steel adsorbent cartridge filled with 150 mg Tenax TA and 200 mg Carbograph 1TD (Markes International Limited, Llantrisant, UK). In addition, blank samples were collected to obtain an estimate of the potential VOC emissions from the metal base, aluminium frames and the polycarbonate chamber inside the growth chambers. Using filtered, VOC-free incoming air may cause an increased diffusion gradient between soil and the chamber headspace, which would lead to a slight overestimation of the emissions. In addition, the potential uptake of VOCs cannot be detected by the used measurement system.

VOCs were analyzed by gas chromatography-mass spectrometry (6850 Network GC system and a 5975C VL MSD with triple axis detector, Agilent, Santa Clara, CA, USA) after thermodesorption at 250°C and cryofocusing at −10°C with a UNITY 2 thermal desorber (Markes, Llantrisant, UK) coupled with a ULTRA 2 autosampler. The compounds were separated using an HP-5 capillary column (50 m × 0.2 mm, film thickness 0.33 μm). Helium was used as the carrier gas. The oven temperature was held at 40°C for 1 min, raised to 210°C at a rate of 5°C min^−1^, and then raised to 250°C at a rate of 20°C min^−1^.

The compounds were identified using standard compounds and the NIST library, and those present in blank samples were omitted from further analysis. The quantification was done using pure standards solutions for α-pinene, borneol, β-myrcene, copaene, trans-β-farnesene, humulene, aromadendrene, δ-cadinene, trans-2-hexenal, cis-3-hexenol, cis-3-hexenyl acetate, 1-octen-3-ol, cis-3-hexenyl butyrate, cis-3-hexenyl isovalerate, and nonanal (Fluka, Buchs, Switzerland). To quantify the compounds without a specific standard, we used a pure standard for as similar compound as possible.

The emission rates were calculated following the procedure outlined in Faubert et al. (2012) taking into account the different soil surface microtopographies in each mesocosm and the additional air volume due to the slightly higher flow rate into than out from the chamber.

### CO_2_ exchange measurements

Carbon dioxide exchange was measured in conjunction with the VOC sampling using an EGM-4 gas monitor (PP Systems, Hitchin, UK). A transparent chamber equipped with a fan was placed on top of the water-filled groove of the aluminium frame and the headspace CO_2_ concentration was recorded for max. 5 min. The chamber was lifted up to air it before repeating the measurement with a darkened chamber. A linear regression of the change in the CO_2_ concentration in light was used as an estimate of net ecosystem exchange (NEE) and that in the dark of the dark ecosystem respiration (R_TOT_). The gross photosynthesis (P_G_) could be derived by subtracting R_TOT_ from NEE.

### Soil microbial biomass and C and N analyses

After the last gas exchange measurements, the hand-sorted and well mixed soil was sampled and analyzed for NH^+^_4_, NO^−^_3_, dissolved organic nitrogen (DON), dissolved organic carbon (DOC), microbial biomass carbon (C_MIC_), and microbial biomass nitrogen (N_MIC_) following standard extraction and fumigation-extraction procedures (Jenkinson and Powlson, 1976; Vance et al., 1987; Rinnan et al., 2008).

Shortly, a 10-g subsample of soil was fumigated with chloroform for 24 h. These and another set of 10-g subsamples were extracted with 50 ml water in a rotary shaker for 1 h following filtration through Whatman GF/D filters. Then, the non-fumigated samples were analyzed for NH^+^_4_ and NO^−^_3_ on a Fiastar 5000 flow injection analyzer (FOSS Tecator, Höganäs, Sweden) and both the fumigated (to determine C_MIC_ and N_MIC_) and non-fumigated (to determine DOC and DON) samples were analyzed for total organic N on the FOSS Fiastar 5000 and for total organic C on the total organic carbon analyzer TOC 5000A (Shimadzu, Kyoto, Japan).

C_MIC_ and N_MIC_ were calculated as the difference in dissolved C and N in the fumigated and the non-fumigated samples. The values were corrected for incomplete extractability by a factor 0.45 for microbial C (Joergensen, 1996) and a factor 0.40 for microbial N (Jonasson et al., 1996).

### Statistical analyses

The data were analyzed for differences between the vegetation manipulation treatments by univariate (single variables, e.g., total BVOC emissions) or multivariate (e.g., vegetation cover percentages) analysis of variance in which the vegetation manipulation and time (when appropriate) were set as fixed factors and the growth chamber as a random factor. When the effect of vegetation manipulation was significant, the data were subjected to Tukey's HSD post hoc tests to identify significant differences between the three treatment levels. The *Deschampsia* and mixed heath mesocosms were analyzed separately, as the data was derived from two separate experiments, and the soil and ecosystem types clearly differed from each other.

## Results

### BVOC emissions and vegetation in the heath mesocosms

The BVOC emissions from the mixed heath mesocosms with intact vegetation cover were composed of sesquiterpenes and non-terpenoid compounds (Table 1). In total, 20 compounds were detected and of these 16 could be identified. The most emitted individual compounds were methyl-2-ethylhexanoate, β-selinene, and 2-methylfuran (Table 1).

**Table 1:** **Emission rates (mean ± SE) of biogenic volatile organic compounds emitted from the mixed heath and *Deschampsia* mesocosms with intact vegetation**.

**Site**	**Compound**	**Emission rate (μg m^−2^ h^−1^)**
Mixed heath^a^		
	α-Copaene	0.30 ± 0.08
	α-Bourbonene	0.01 ± 0.01
	trans-Caryophyllene	0.08 ± 0.02
	γ-curcumene	0.09 ± 0.03
	Aromadendrene	0.07 ± 0.02
	Humulene	0.22 ± 0.05
	Valencene	0.10 ± 0.04
	β-Selinene	1.94 ± 0.57
	α-Selinene	0.81 ± 0.28
	δ-Cadinene	0.02 ± 0.01
	Selina-3,7(11)-diene	0.14 ± 0.06
	Unidentified SQT	0.01 ± 0.01
	Unidentified SQT	0.04 ± 0.01
	Unidentified SQT	0.10 ± 0.05
	*Total sesquiterpenes*	*3.94 ± 1.05*
	2-Methylfuran	1.63 ± 0.40
	Methoxy-phenyl-oxime	0.39 ± 0.14
	Styrene	0.21 ± 0.07
	Methyl 2-ethylhexanoate	2.36 ± 0.37
	Benzenepropanol	0.04 ± 0.02
	Unidentified compound	1.76 ± 0.43
	*Total other compounds*	*6.39 ± 1.10*
	*Total BVOCs*	*10.33 ± 1.39*
*Deschampsia*		
	cis-Ocimene	0.05 ± 0.05
	*Total monoterpenes*	*0.05* ± *0.05*
	Methoxy-phenyl-oxime	0.55 ± 0.27
	Phenol	0.09 ± 0.09
	3-Hexenyl acetate	2.35 ± 1.50
	Methyl 2-ethylhexanoate	0.18 ± 0.07
	3-methylheptylacetate	0.02 ± 0.02
	*Total other compounds*	*3.19 ± 1.49*
	*Total BVOCs*	*3.24 ± 1.48*

a *The mixed heath was dominated by evergreen and deciduous dwarf shrubs (see text for details)*.

*SQT, sesquiterpene*.

The vegetation cover in the mixed heath mesocosms was not significantly different before the manipulations were performed (*P* > 0.5, MANOVA), so any differences between the mesocosm types should not be due to different vegetation. Averaged across all mesocosms, the cover percentages of the different species were 69 ± 3% for *Empetrum hermaphroditum*, 10 ± 5% for *Rhododendron lapponicum*, 3 ± 1% for *Andromeda polifolia*, and *Vaccinium uliginosum*, 2 ± 1% for graminoids, and 4 ± 1% for bryophytes. Furthermore, the total belowground biomass was similar in all mesocosms types (*P* > 0.35, ANOVA; that for soil mesocosms harvested when manipulations started and for the other treatments at the end of the experiment). Leaf and stem biomass for each species is shown in Table S1 in Supplementary Material.

The BVOC emissions from the *Deschampsia* mesocosms consisted of non-terpenoid compounds, in addition to the low emission of the monoterpene cis-ocimene (Table 1). The compound that was emitted in the highest quantity was 3-hexenyl acetate.

The total *Deschampsia* shoot biomass was 1.2 ± 0.1 kg m^−2^ in the root and soil mesocosms at the time of vegetation manipulations, and 1.8 ± 0.2 kg m^−2^ in the control mesocosms at the end of the experiment (*P* < 0.01, ANOVA followed by Tukey's HSD). The total belowground biomass was 3.2 ± 1.0 kg m^−2^ in the control mesocosms and not significantly different in the other mesocosms types (*P* > 0.2, ANOVA).

### Effects of vegetation manipulations on BVOC emissions

**Figure 2:**
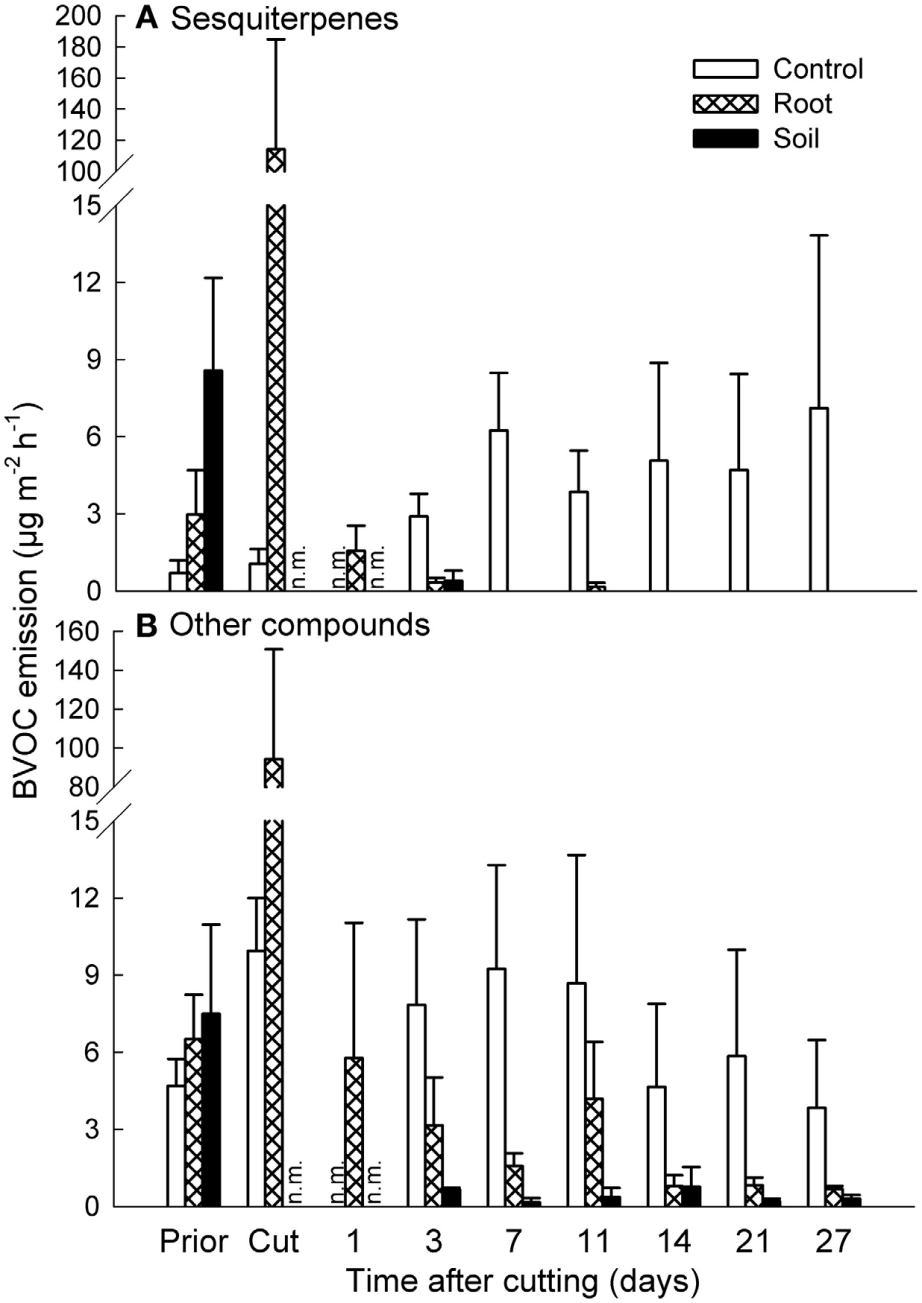
**Effect of experimental manipulations on biogenic volatile organic compound (BVOC) emissions from mixed heath mesocosms.** The total emissions of **(A)** sesquiterpenes and **(B)** other BVOCs (mean + SE, *n* = 3) with intact vegetation (control), cut aboveground vegetation (root mesocosms), and all vegetation removed (soil mesocosms) prior to cutting (Prior), directly after cutting (Cut) and after cutting at different time intervals. n.m., not measured.

Cutting of aboveground vegetation caused high emission bursts from the mesocosms (Figures 1, 2). For mixed heath mesocosms, the total emission of sesquiterpenes increased from 3.0 ± 1.7 μg m^−2^ h^−1^ (mean ± SE, root mesocosms) right before cutting to 114 ± 71 μg m^−2^ h^−1^ measured within an hour after cutting. The following day the total sesquiterpene emissions decreased to 1.6 ± 1.0 μg m^−2^ h^−1^. The total emission of other compounds changed from 6.5 ± 1.7 μg m^−2^ h^−1^ before to 94 ± 56 μg m^−2^ h^−1^ immediately following cutting, and then to 5.8 ± 5.3 μg m^−2^ h^−1^ the day after cutting (Figure 2). The increase in emission rates owed mainly to the induced emission of various C8-compounds (e.g., 1-octene, 1,3-octadiene, 2-octen-1-ol, and 1-octanone) and sesquiterpenes, most of which could not be identified to compound level (Table 2; Table S2 in Supplementary Material).

**Table 2:** **List of biogenic volatile organic compounds induced by cutting of aboveground vegetation**.

**Mixed heath mesocosms**	***Deschampsia* mesocosms**
1-Octene	1,3-Octadiene
1,3-Octadiene	1,3,5-Octatriene
2-Octen-1-ol	2-Octen-1-ol
2-Octanone	2-Octanone
3-Octanol	β-Myrcene
Pentyl propanate	2-methylenebornane
trans-Caryophyllene	3-Octanol
γ-curcumene	Pentyl propanate
Aromadendrene	Geosmin
α-Elemene	Unidentified MT
β-Selinene	
α-Selinene	
α-Guaiene	
Selina-3,7(11)-diene	
Unidentified SQT	

*The emission of the listed compounds increased at least by one order of magnitude directly after cutting. See Tables S2, S3 in Supplementary Material for emission rates*.

*SQT, sesquiterpene; MT, monoterpene*.

**Figure 3:**
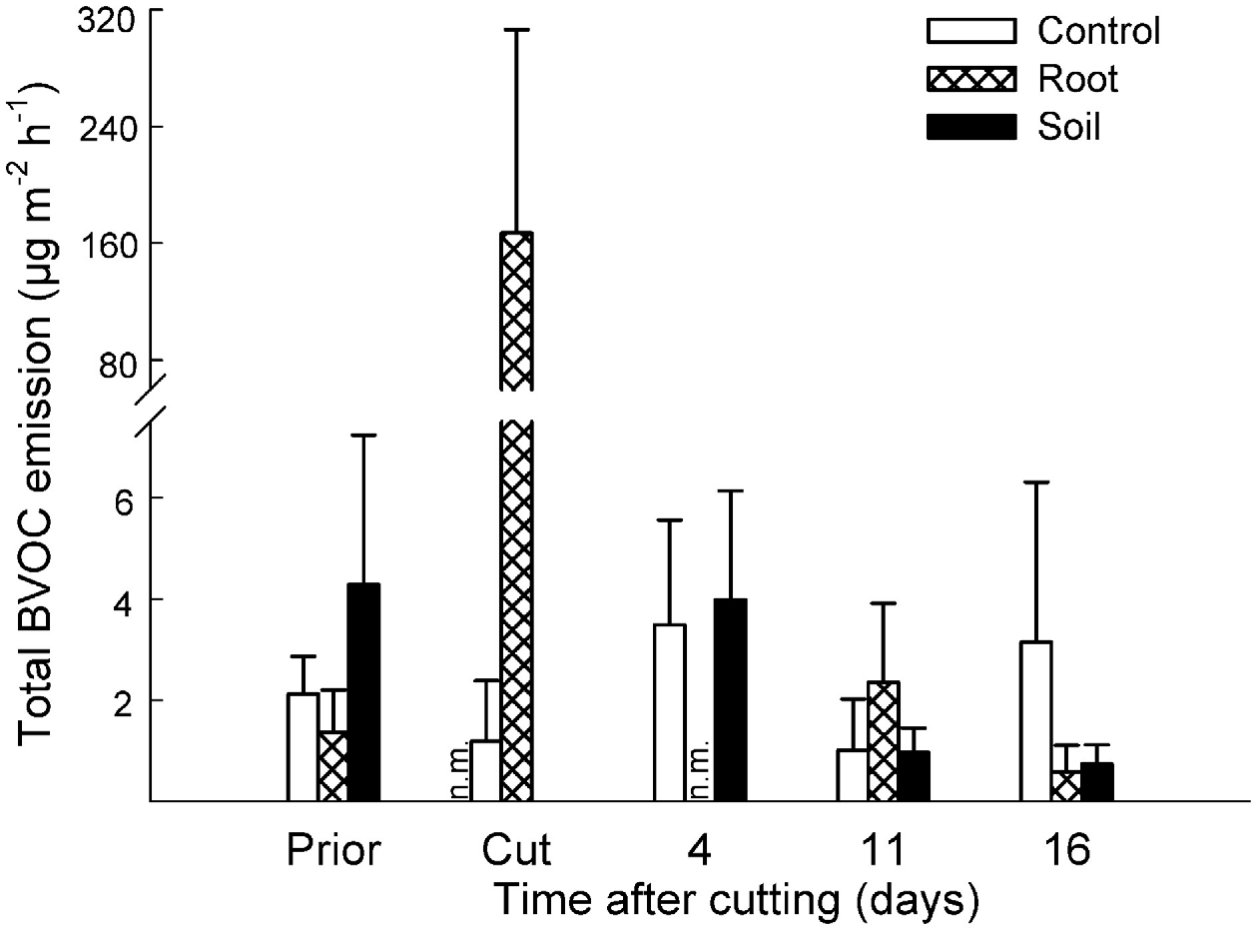
**Effect of experimental manipulations on biogenic volatile organic compound (BVOC) emissions from *Deschampsia* mesocosms.** The total emissions of BVOCs (mean + SE, *n* = 3) with intact vegetation (control), cut aboveground vegetation (root mesocosms) and all vegetation removed (soil mesocosms) prior to cutting (Prior), directly after cutting (Cut) and after cutting at different time intervals. n.m., not measured.

In *Deschampsia* mesocosms, the constitutive emissions were composed of non-terpenoid compounds, and therefore the grouping of compounds was omitted. The total BVOC emissions increased from 1.4 ± 0.8 μg m^−2^ h^−1^ prior to cutting to 167 ± 139 μg m^−2^ h^−1^ after the cutting of aboveground vegetation (Figure 3). Cutting induced an emission of 24 compounds, various mono- and sesquiterpenes and C8 compounds, of which ten had an emission rate above 1 μg m^−2^ h^−1^ (Table 2; Table S3 in Supplementary Material).

The effects of vegetation cutting on the emissions had ceased by the following day (Figure 2, Tables S2, S3 in Supplementary Material). During the rest of the experiment, the emissions from the control mixed heath mesocosms were considerably higher than the emissions from root and soil mesocosms (Figure 2). Sesquiterpenes were solely emitted from the mesocosms with aboveground vegetation at the rate of 5.4 μg m^−2^ h^−1^ averaged across the measurement dates (Figure 2A). The emissions of other compounds were 4 and 18 times higher from the control mesocosms than from the root and soil mesocosms, respectively (Figure 2B).

In *Deschampsia* mesocosms, there were no significant differences between the vegetation treatments in the period 4–16 days after cutting (Figure 3).

### CO_2_ exchange

The mesocosms from both heath locations were net sources of CO_2_ to the atmosphere with lower gross photosynthesis (P_G_) rate than dark ecosystem respiration (R_TOT_) rate during the experiment. Cutting of the aboveground vegetation or removing the belowground biomass did not cause any immediate alterations in CO_2_ exchange, and only the data from after the established treatments are shown.

**Figure 4:**
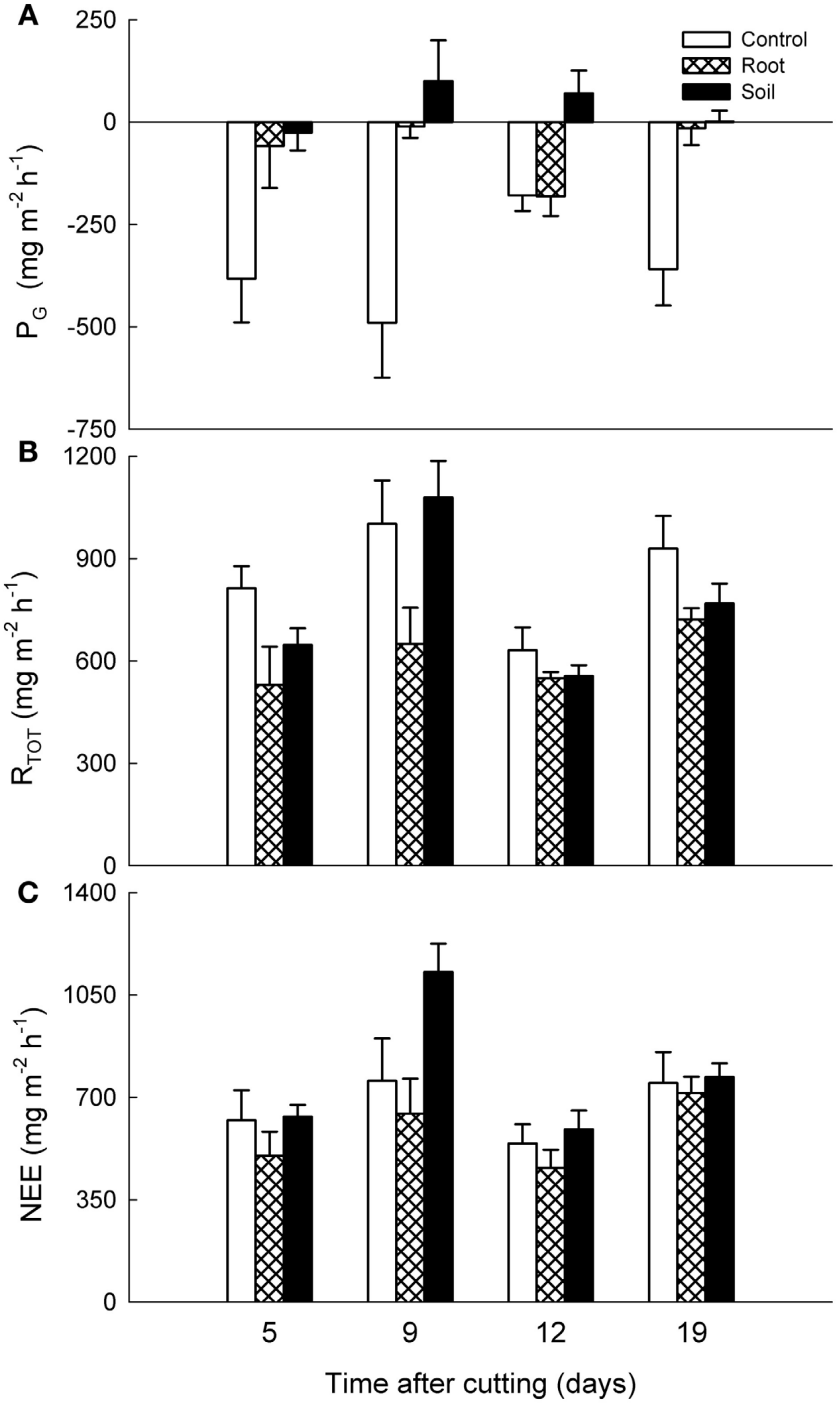
**Effects of experimental manipulations on CO_2_ exchange in mixed heath mesocosms.** Mean (+ SE, *n* = 3) **(A)** gross photosynthesis (P_G_), **(B)** dark ecosystem respiration (R_TOT_) and **(C)** net ecosystem exchange (NEE) with intact vegetation (control), cut aboveground vegetation (root mesocosms), and all vegetation removed (soil mesocosms) as function of time after cutting. Positive values denote carbon loss and negative values carbon uptake into the system.

**Figure 5:**
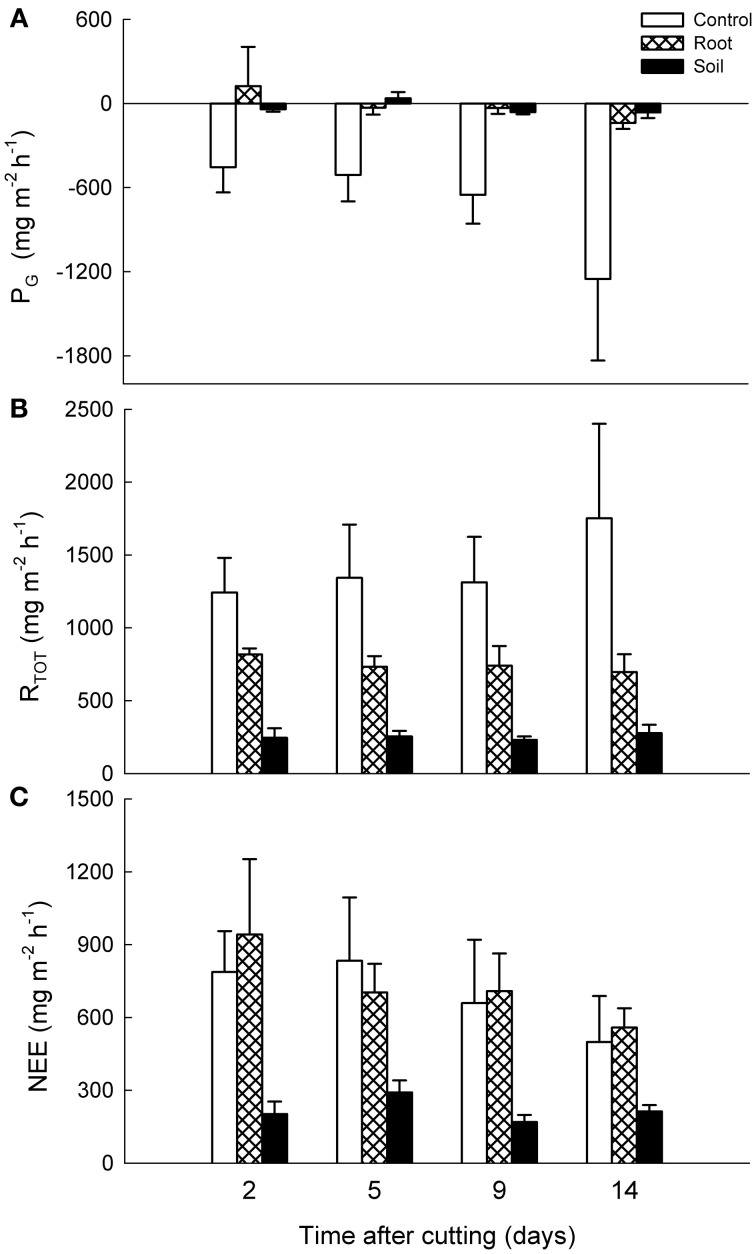
**Effects of experimental manipulations on CO_2_ exchange in *Deschampsia* mesocosms.** Mean (+ SE, *n* = 3) **(A)** gross photosynthesis (P_G_), **(B)** dark ecosystem respiration (R_TOT_), and **(C)** net ecosystem exchange (NEE) with intact vegetation (control), cut aboveground vegetation (root mesocosms) and all vegetation removed (soil mesocosms) as function of time after cutting. Positive values denote carbon loss and negative values carbon uptake into the system.

There were no statistically significant differences between the vegetation treatments in NEE, P_G_, or R_TOT_ of the mixed heath mesocosms, although the control mesocosms showed some gross carbon assimilation while the root and soil mesocosms did not (Figure 4). In contrast, for *Deschampsia* mesocosms the NEE was significantly lower in the soil mesocosms than in the control and root mesocosms (*P* < 0.001; Figure 5). For R_TOT_, all treatments differed significantly from each other, with highest total respiration in the control mesocosms followed by root and soil mesocosms. Carbon uptake into the system as a result of P_G_ was naturally highest in the control mesocosms and significantly lower in the root and soil mesocosms.

### Concentrations of C and N in soil and microbial biomass

In the mixed heath soil, the concentrations of NH^+^_4_, NO^−^_3_, DOC, and DON in the soil mesocosms were 43–67% lower than the concentrations in the control mesocosms, although the difference for NH^+^_4_ was only marginally significant (0.05 < *P* < 0.1, Tukey's HSD; Table 3). There were no significant differences in C_MIC_ and N_MIC_ concentrations between the mesocosms types (*P* > 0.3, ANOVA).

**Table 3:** **Concentrations of NH^+^_4_, NO^−^_3_, dissolved organic carbon (DOC), dissolved organic nitrogen (DON), microbial biomass carbon (C_MIC_), and microbial biomass nitrogen (N_MIC_) in soil from the mixed heath and *Deschampsia* mesocosms**.

	**Mixed heath mesocosms**			***Deschampsia* mesocosms**		
	**Control**	**Root**	**Soil**	**Control**	**Root**	**Soil**
NH^+^_4_-N (μg g^−1^ SOM)	3.6 ± 0.6	3.2 ± 0.3	1.8 ± 0.1	31 ± 16	137 ± 41	103 ± 54
NO^−^_3_-N (μg g^−1^ SOM)	0.81 ± 0.16^a^	0.61 ± 0.10^a, b^	0.27 ± 0.06^b^	80 ± 23	260 ± 67	159 ± 134
DOC (mg g^−1^ SOM)	1.6 ± 0.3^a^	1.4 ± 0.1^a, b^	0.9 ± 0.1^b^	2.6 ± 0.3^a^	2.5 ± 0.1^a^	4.1 ± 0.4^b^
DON (μg g^−1^ SOM)	125 ± 23^a^	110 ± 9^a, b^	65 ± 4^b^	565 ± 62^a^	843 ± 92^b^	1255 ± 58^c^
C_MIC_ (mg g^−1^ SOM)	5.8 ± 0.7	5.1 ± 0.3	5.3 ± 0.2	6.4 ± 1.1	5.8 ± 0.7	5.9 ± 0.6
N_MIC_ (μg g^−1^ SOM)	442 ± 57	397 ± 18	430 ± 28	569 ± 183	548 ± 119	570 ± 30

*The mesocosms had intact vegetation (control), had cut aboveground vegetation (root mesocosms) or all vegetation removed (soil mesocosms). Soil was analysed after the last gas exchange measurements*.

*The values are mean ± SE*.

*The different letters (a, b, c) indicate statistically significantly differences between the mesocosms types within a location at 95% significance level (Tukey's HSD test)*.

In *Deschampsia* soil, the variation was higher, and the only statistically significant differences were the higher concentrations of DOC and DON in the soil mesocosms than in the control and root mesocosms (*P* < 0.05, Tukey's HSD; Table 3). For DON, the concentration was lowest in the control, 50% higher in the root mesocosms (*P* < 0.01, Tukey's HSD; Table 3) and more than doubled in the soil mesocosms (*P* < 0.001, Tukey's HSD).

## Discussion

### Constitutive BVOC emissions from heath ecosystems outside the growing season

The two heath ecosystems under investigation emitted contrasting BVOCs from different sources. While the *Deschampsia*-dominated temperate heath was characterized by low BVOC emissions from vegetation, the subarctic mixed heath had clearly vegetation-dominated emissions, despite the vegetation being less active due to onset of autumn, as shown by the low gross photosynthesis rates. The gross photosynthesis rate in the mixed heath mesocosms was in the same range as that measured *in situ* before the growing season start in May (Nielsen, et al., unpublished data). Despite the plants being less active, both vegetation types reacted with strongly induced emissions to cutting of the aboveground vegetation. This is most likely because the BVOCs released upon cutting originate from storage structures, such as resin ducts or glandular trichomes, and not from *de novo* synthesis (Holopainen and Gershenzon, 2010).

A characteristic feature for the emissions from the mixed heath mesocosms with nearly 70% *E. hermaphroditum* cover was the lack of isoprene and monoterpenes, and the higher amount and variety of sesquiterpene emissions. This finding is in line with the results of Faubert et al. (2012) who found that *E. hermaphroditum* was an important sesquiterpene source for the mountain birch forest floor emissions measured during the growing season. The total BVOC emission rate of the mixed heath mesocosms, 10.33 μg m^−2^ h^−1^, was in the same range as the emission rates during the growing season from *in situ* measurements in Abisko: 10.9–14.61 μg m^−2^ h^−1^ for a mixed wet heath (Tiiva et al., 2008; Faubert et al., 2010) and 3.5–45 μg m^−2^ h^−1^ for an *E. hermaphroditum*-dominated forest floor (Faubert et al., 2012), depending on the year. The emission rates of the present experiment may be somewhat understated due to the light intensity in the growth chambers being lower than for open ecosystems under field conditions in full sunlight. However, the light intensity was similar to shaded or clouded conditions in a forest understory (Olsrud and Michelsen, 2009). The lacking isoprene and monoterpene emissions are likely due to that the vegetation has been without active *de novo* synthesis of BVOCs in the photosynthesizing green leaves. It appears, however, that despite lacking photosynthetic activity, the evergreen plants are releasing BVOCs at a temperature of about 13.6°C.

The constitutive emissions from the *Deschampsia* mesocosms lacked all terpenoids. This is in agreement with an earlier qualitative assessment of emissions from *D*. *flexuosa*, which indicated no isoprene or monoterpene emissions (Hewitt and Street, 1992). In fact, there were no significant differences in emissions from the *Deschampsia* mesocosms among control, root and soil mesocosms, suggesting that the plants were a minor source for the ecosystem-level emissions in these off-season measurements, except immediately after cutting.

### Source of the emitted BVOCs

Most BVOCs emitted from the mixed heath mesocosms were clearly produced by plants as their emission ceased after the aboveground vegetation was removed by cutting. Further, the net emissions originating below soil surface appeared to be mainly derived from roots and rhizomes. It should be taken into account that by cutting the vegetation also the transport of assimilates into belowground plant parts ceases and root exudation rates are likely to decrease, thereby reducing potential BVOC release both from belowground plant parts and soil microbial activity fuelled by root exudates. However, we assume that assimilate transport within the plants is minimal outside the main growth period.

The emissions of 2-methylfuran and methyl-2-ethylhexanoate, both of which can be used as indicators of microbial growth on construction materials (e.g., Korpi et al., 2009), were halved by cutting and reduced to below the detection limit when belowground plant parts were removed from the soil. This suggests that these compounds could originate from decomposition of root exudates released from living plants. Different furans are widely emitted from soils (Leff and Fierer, 2008; McNeal and Herbert, 2009), and therefore their absence in the soil mesocosm emissions was surprising. However, these compounds were also not detected in the emissions from a mountain birch forest floor with removed aboveground vegetation cover (Faubert et al., 2012).

The only compound emitted from all the mesocosm types in both heath ecosystems, including the soil mesocosms, was methoxy-phenyl-oxime. This N-containing aromatic compound has been previously observed to be emitted from soil (McNeal and Herbert, 2009). It is likely to originate from microbial activity as it has been reported to be an antifungal volatile emitted by *Bacillus subtilis* bacteria isolated from soil (Liu et al., 2008), to be released from myxobacteria during fermentation (Xu et al., 2011) and to be synthesized by an *in vitro* system consisting of the ectomycorrhizal fungus *Tuber borchii* and the *Tilia americana* L. plant roots (Menotta et al., 2004).

The lack of net emissions from soil may owe to a lack of microbial BVOC production in the absence of decaying belowground plant biomass and root exudates or stimulated uptake of BVOCs by soil microorganisms (Owen et al., 2007; Ramirez et al., 2010) as a result of increased aeration of soil due to the hand-sorting of the soil upon removal of roots and rhizomes. The hand-sorting received by the soil mesocosms caused a side-effect to this treatment in the form of broken physical structure and increased aeration of microsites within soil. We estimate that the stimulative effect on microbial uptake of BVOCs is more important than the suppressive effect on gross microbial BVOC production, but these two processes cannot be separated in the present study.

In the mixed heath mesocosms, the removal of belowground plant parts significantly reduced the concentrations of DOC and DON in the soil, while there was no difference in the CO_2_ exchange rates between the vegetation manipulation treatments. In *Deschampsia* mesocosms, in contrast, the concentrations of DOC and DON were significantly higher and the loss of CO_2_ decreased to less than half of the other mesocosm types after the removal of belowground plant parts. Hence, in the more sandy *Deschampsia* dominated system, mineralization of dissolved organic compounds was partially dependent upon the presence of roots, while microbial turnover of dissolved organic compounds continued in the absence of roots in the mixed heath, possibly due to higher fungal dominance (microbial C/N ratio) and higher soil organic matter content.

Other BVOCs potentially emitted are carbonyls, but our measurement setup could not be used to measure these compounds, because of breakthrough of these light molecules through the used adsorbents. Carbonyls were the compounds emitted at highest rates by cultured boreal forest soil fungi, as highlighted by a proton transfer reaction-mass spectrometer (PTR-MS) study by Bäck et al. (2010). Other PTR-MS studies have also shown that the short-chain carbonyls, such as methanol and acetone, are compounds emitted at high rates from litter and soil (Ramirez et al., 2010; Gray and Fierer, 2012).

### Emissions induced by vegetation cutting

Cutting the aboveground vegetation caused large emission bursts, which is a common observation in response to mechanical wounding of plants (Fall et al., 1999; Loreto et al., 2006; Brilli et al., 2011). However, in contrast with the earlier studies reporting high emissions of C6 compounds (so called green leaf volatiles, GLVs) (Fall et al., 1999; Loreto et al., 2006; Brilli et al., 2011), the induced emissions here mainly consisted of C8-ketones, C8-alcohols and sesquiterpenes. The C8-compounds such as 1-octen-3-ol, 3-octanol, 3-octanone have common precursors with the commonly observed GLVs, namely linoleic and linolenic acid (Wurzenberger and Grosch, 1982; Hatanaka, 1993).

Some C8-compounds have been reported to be emitted from cabbage leaves in response to mechanical damage and herbivore wounding (Mattiacci et al., 1994). However, *Deschampsia cespitosa* has been shown to emit these compounds both with and without mechanical damage or jasmonic acid application (Watkins et al., 2006). Another possibility is that the C8-compounds were released from fungal endophytes living within the leaves (Rosa et al., 2009) and roots (Tejesvi et al., 2012) of *Deschampsia*. Both C8-compounds and geosmin, which was also induced by cutting of *Deschampsia*, are common volatiles produced by microorganisms, especially fungi (Korpi et al., 2009). Also cyanobacteria are known to emit C8-compounds (Schulz and Dickschat, 2007), and it is possible that the cyanobacteria living within mosses are a source of part of the induced emissions especially in the mixed heath mesocosms from which the moss cover was removed and the emissions increased by cutting.

Studies using online monitoring by PTR-MS of BVOCs produced in connection with mechanical wounding of plants have shown that the instantaneous release of C6 aldehydes originating from membrane rupture and the consequent oxidation of unsaturated fatty acids shifts to the production of alcohols and acetates within a couple of minutes from wounding (Fall et al., 1999; Brilli et al., 2011). In this study, the BVOC measurement was started within 10 min from finishing the cutting, which means that the compounds immediately released from the ruptured plant tissues were most probably not caught by our measurements.

To conclude, the net BVOC emissions from the belowground part of these well-drained heath ecosystems do not significantly contribute to the ecosystem emissions, at least during late autumn when the vegetation is not fully active. We recommend that future studies attempting to connect soil BVOC exchange with soil microbiology would use methods that can separate production and uptake of BVOCs, for example by PTR-MS measurements, and combine these with analyses of microbial community structure and function e.g., by DNA- and RNA-based techniques. In general, the total BVOC emissions from the mixed heath were in the same range as the emissions measured in a similar ecosystem with active vegetation and peaking biomass. This finding highlights the importance of taking into account the off-season period, also for the northern areas. The ecosystem emissions are likely to be momentarily or periodically increased by the production of induced volatiles as a result of grazing by mammals or insect herbivory. Especially the latter can have a significant impact on subarctic heath BVOC emissions as large-scale insect outbreaks are expected to become more frequent in a warmer climate (Arneth and Niinemets, 2010).

### Conflict of interest statement

The authors declare that the research was conducted in the absence of any commercial or financial relationships that could be construed as a potential conflict of interest.
