# Supplementary material for: Off-season biogenic volatile organic compound emissions from heath mesocosms: responses to vegetation cutting
Source: Front Microbiol. 2013 Aug 15;4:224. doi: 10.3389/fmicb.2013.00224 (PMC3744039; doi:10.3389/fmicb.2013.00224)
Supplement: Supplementary file 1 [file DataSheet1.DOCX]

**Supplementary material**

**Off-season biogenic volatile organic compound emissions from heath mesocosms: responses to vegetation cutting**

*Riikka Rinnan, Diana Gierth, Merete Bilde, Thomas Rosenørn and Anders Michelsen

**Table S1** Dry biomass (mean ± SE, *n* = 3) for the plant species in the mixed heath mesocosms

| Species | Biomass (g m^-2^) | Control | | Root | | Soil | |
| --- | --- | --- | --- | --- | --- | --- | --- |
| *Empetrum hermaphroditum* | Leaves | 271 | ± 28 | 216 | ± 33 | 242 | ± 9 |
|  | Stems | 302 | ± 14 | 199 | ± 27 | 236 | ± 16 |
| *Andromeda polifolia* | Leaves | 33 | ± 17 | 25 | ± 6 | 26 | ± 9 |
|  | Stems | 34 | ± 7 | 33 | ± 8 | 40 | ± 8 |
| *Rhododendron lapponicum* | Leaves | 4.3 | ± 2.3 | 12 | ± 4 | 39 | ± 11 |
|  | Stems | 42 | ± 19 | 58 | ± 27 | 130 | ± 28 |
| *Vaccinium uliginosum* | Leaves | 0.3 | ± 0.1 | 3.5 | ± 0.3 | 4.8 | ± 0.7 |
|  | Stems | 43 | ± 7.5 | 25 | ± 12 | 63 | ± 15 |
| *Arctostaphylos alpina* | Shoots | 2.1 | ± 1.1 | 3.1 | ± 3.0 | 7.0 | ± 4.9 |
| *Tofieldia pusilla* | Shoots | 9.3 | ± 3.1 | 4.5 | ± 3.2 | 4.9 | ± 2.7 |
| *Carex vaginata* | Shoots | 0.0 | ± 0.0 | 3.3 | ± 3.3 | 2.8 | ± 2.8 |
| Mosses | (green parts) | 23.3 | ± 2.8 | 60 | ± 9 | 56 | ± 15 |
| Lichens | (total) | 23.0 | ± 3.0 | 32 | ± 8 | 35 | ± 18 |

**Table S2** Emissions of biogenic volatile organic compounds (mean ± SE, *n* = 3) from the same mixed heath mesocosms before, immediately after and 1 day after removing the aboveground vegetation by cutting

|  | Before cutting | | After cutting | | 1 day after cutting | |
| --- | --- | --- | --- | --- | --- | --- |
|  | Mean | SE | Mean | SE | Mean | SE |
| 3-methylhexane | 0.0 | 0.0 | 0.0 | 0.0 | 2.8 | 2.8 |
| Methylcyclohexane | 0.0 | 0.0 | 0.0 | 0.0 | 1.9 | 1.9 |
| 1-Octene | 0.0 | 0.0 | 2.5 | 1.1 | 0.0 | 0.0 |
| 1,3-Octadiene | 0.0 | 0.0 | 85.3 | 55.0 | 0.0 | 0.0 |
| 1,3,5-Octatriene | 0.0 | 0.0 | 0.7 | 0.3 | 0.0 | 0.0 |
| Furyl ethyl ketone | 0.0 | 0.0 | 0.1 | 0.1 | 0.0 | 0.0 |
| 2-Methylfuran | 0.4 | 0.4 | 0.5 | 0.5 | 0.6 | 0.4 |
| 2-Octen-1-ol | 0.0 | 0.0 | 32.2 | 14.9 | 0.0 | 0.0 |
| 2-Octanone | 0.0 | 0.0 | 54.6 | 25.6 | 0.0 | 0.0 |
| Unidentified compound | 0.0 | 0.0 | 0.9 | 0.3 | 0.0 | 0.0 |
| 3-Octanol | 0.0 | 0.0 | 4.7 | 2.2 | 0.0 | 0.0 |
| Pentyl propanate | 0.0 | 0.0 | 1.5 | 0.8 | 0.0 | 0.0 |
| Butyl furan | 0.0 | 0.0 | 0.1 | 0.1 | 0.0 | 0.0 |
| Unidentified compound | 0.5 | 0.5 | 0.0 | 0.0 | 0.0 | 0.0 |
| Methyl 2-ethylhexanoate | 3.7 | 2.6 | 0.8 | 0.2 | 0.5 | 0.2 |
| 2-Octenal | 0.0 | 0.0 | 0.1 | 0.1 | 0.0 | 0.0 |
| Unidentified SQT | 0.0 | 0.0 | 0.2 | 0.2 | 0.0 | 0.0 |
| Unidentified SQT | 0.0 | 0.0 | 0.1 | 0.1 | 0.0 | 0.0 |
| α-Muurolene | 0.0 | 0.0 | 0.1 | 0.1 | 0.0 | 0.0 |
| α-Copaene | 0.1 | 0.1 | 0.2 | 0.2 | 0.0 | 0.0 |
| α-Bourbonene | 0.0 | 0.0 | 0.3 | 0.2 | 0.0 | 0.0 |
| Unidentified SQT | 0.0 | 0.0 | 0.1 | 0.1 | 0.0 | 0.0 |
| Unidentified SQT | 0.0 | 0.0 | 0.2 | 0.1 | 0.0 | 0.0 |
| Unidentified SQT | 0.0 | 0.0 | 0.3 | 0.3 | 0.0 | 0.0 |
| Unidentified SQT | 0.0 | 0.0 | 0.1 | 0.1 | 0.0 | 0.0 |
| trans-Caryophyllene | 0.0 | 0.0 | 2.4 | 1.2 | 0.0 | 0.0 |
| γ-curcumene | 0.0 | 0.0 | 1.0 | 0.4 | 0.0 | 0.0 |
| Germacrene D | 0.0 | 0.0 | 0.1 | 0.1 | 0.0 | 0.0 |
| Trans-β-Farnesene | 0.0 | 0.0 | 0.2 | 0.2 | 0.0 | 0.0 |
| Aromadendrene | 0.0 | 0.0 | 1.9 | 1.1 | 0.0 | 0.0 |
| Humulene | 0.1 | 0.1 | 7.9 | 3.3 | 0.0 | 0.0 |
| α-Elemene | 0.0 | 0.0 | 3.8 | 1.9 | 0.0 | 0.0 |
| Unidentified SQT | 0.0 | 0.0 | 0.7 | 0.4 | 0.0 | 0.0 |
| Valencene | 0.0 | 0.0 | 0.6 | 0.1 | 0.0 | 0.0 |
| β-Selinene | 2.8 | 2.3 | 123.9 | 68.5 | 1.1 | 0.7 |
| α-Selinene | 1.3 | 1.1 | 60.5 | 33.0 | 0.4 | 0.2 |
| Unidentified SQT | 0.0 | 0.0 | 0.3 | 0.3 | 0.0 | 0.0 |
| Unidentified SQT | 0.0 | 0.0 | 0.6 | 0.3 | 0.0 | 0.0 |
| δ-Cadinene | 0.0 | 0.0 | 0.8 | 0.5 | 0.0 | 0.0 |
| α-Guaiene | 0.1 | 0.1 | 2.4 | 1.2 | 0.0 | 0.0 |
| Unidentified SQT | 0.1 | 0.1 | 7.4 | 4.0 | 0.0 | 0.0 |
| Selina-3,7(11)-diene | 0.1 | 0.1 | 4.5 | 2.9 | 0.0 | 0.0 |
| Unidentified SQT | 0.0 | 0.0 | 2.5 | 2.1 | 0.0 | 0.0 |
| Unidentified SQT | 0.0 | 0.0 | 0.3 | 0.2 | 0.0 | 0.0 |

SQT, sesquiterpene

**Table S3** Emissions of biogenic volatile organic compounds (mean ± SE, *n* = 3) from the same *Deschampsia* mesocosms before, immediately after and 1 day after removing the aboveground vegetation by cutting

|  | Before cutting | | After cutting | | 1 day after cutting^a^ | | |
| --- | --- | --- | --- | --- | --- | --- | --- |
|  | Mean | SE | Mean | SE | Mean | SE |  |
| 1-Octene | 0.0 | 0.0 | 0.9 | 0.8 | 0.0 | 0.0 |  |
| Hexanal | 0.0 | 0.0 | 0.9 | 0.6 | 0.0 | 0.0 |  |
| 1,3-Octadiene | 0.0 | 0.0 | 3.7 | 3.4 | 0.0 | 0.0 |  |
| 1,3,5-Octatriene | 0.0 | 0.0 | 2.1 | 2.1 | 0.0 | 0.0 |  |
| 3-Heptanone | 0.0 | 0.0 | 0.7 | 0.7 | 0.0 | 0.0 |  |
| Methoxy-phenyl-oxime | 0.1 | 0.1 | 0.3 | 0.1 | 0.1 | 0.1 |  |
| Phenol | 0.0 | 0.0 | 0.4 | 0.4 | 0.0 | 0.0 |  |
| 2-Octen-1-ol | 0.0 | 0.0 | 16.7 | 16.1 | 0.0 | 0.0 |  |
| 2-Octanone | 0.0 | 0.0 | 115.3 | 96.0 | 0.0 | 0.0 |  |
| β-Myrcene | 0.0 | 0.0 | 1.7 | 1.7 | 0.0 | 0.0 |  |
| 2-methylenebornane | 0.0 | 0.0 | 4.5 | 3.5 | 0.0 | 0.0 |  |
| 3-Octanol | 0.0 | 0.0 | 10.1 | 9.7 | 0.0 | 0.0 |  |
| Pentyl propanate | 0.0 | 0.0 | 2.1 | 2.1 | 0.0 | 0.0 |  |
| Unidentified MT | 0.0 | 0.0 | 0.2 | 0.2 | 0.0 | 0.0 |  |
| Benzylchloride | 0.0 | 0.0 | 0.3 | 0.3 | 0.0 | 0.0 |  |
| Methyl 2-ethylhexanoate | 0.4 | 0.1 | 0.2 | 0.2 | 0.5 | 0.5 |  |
| cis-Ocimene | 0.6 | 0.6 | 0.4 | 0.4 | 0.0 | 0.0 |  |
| Unidentified MT | 0.0 | 0.0 | 0.7 | 0.3 | 0.0 | 0.0 |  |
| Undecane | 0.0 | 0.0 | 0.4 | 0.0 | 0.0 | 0.0 |  |
| Unidentified MT | 0.0 | 0.0 | 1.6 | 0.7 | 0.0 | 0.0 |  |
| 2-Methylisoborneol | 0.0 | 0.0 | 0.8 | 0.2 | 0.0 | 0.0 |  |
| 2-Methyl-2-bornene | 0.0 | 0.0 | 0.3 | 0.2 | 0.0 | 0.0 |  |
| Unidentified compound | 0.0 | 0.0 | 0.4 | 0.2 | 0.0 | 0.0 |  |
| Unidentified compound | 0.0 | 0.0 | 0.4 | 0.2 | 0.0 | 0.0 |  |
| Geosmin | 0.0 | 0.0 | 1.1 | 0.2 | 0.0 | 0.0 |  |
| Unidentified SQT | 0.0 | 0.0 | 0.1 | 0.1 | 0.2 | 0.2 |  |
| Unidentified SQT | 0.1 | 0.1 | 0.2 | 0.2 | 0.0 | 0.0 |  |
| Humulene | 0.0 | 0.0 | 0.2 | 0.2 | 0.0 | 0.0 |  |

^a^ *n* = 1

MT, monoterpene; SQT, sesquiterpene
